# Pan-Immune-Inflammation Value as a Predictor of Long-Term Outcomes in Patients with Urothelial Carcinoma of the Bladder: A Pilot Study

**DOI:** 10.3390/curroncol32100534

**Published:** 2025-09-24

**Authors:** Ali Erhan Eren, Asim Armagan Aydin, Eren Erdi Aksaray, Arda Durak, Ahmet Unlu, Mahmut Ekrem Islamoglu, Banu Ozturk, Mustafa Yildiz

**Affiliations:** 1 Department of Urology, University of Health Sciences Antalya Education and Research Hospital, Antalya 07100, Turkey; aksarayerenerdi@gmail.com (E.E.A.); ardadurakk7@gmail.com (A.D.); meislamoglu@gmail.com (M.E.I.); 2 Department of Clinical Oncology, University of Health Sciences Antalya Education and Research Hospital, Antalya 07100, Turkey; drarmaganaydin@gmail.com (A.A.A.); md.ahmetunlu@gmail.com (A.U.); drbanutr@yahoo.com (B.O.); drmyildiz@yahoo.com (M.Y.)

**Keywords:** urothelial carcinoma, bladder cancer, pan-immune-inflammation value, non-muscle invasive, muscle invasive, survival, biomarker, prognosis

## Abstract

Background: Urothelial carcinoma of the bladder (UCB) demonstrates considerable heterogeneity, with markedly varying outcomes between non–muscle-invasive bladder cancer (NMIBC) and muscle-invasive bladder cancer (MIBC). The pan-immune-inflammation value (PIV), derived from routine hematological parameters, has emerged as a novel biomarker reflecting systemic inflammation and immune dysregulation. This pilot, exploratory analysis evaluated the prognostic relevance of the PIV in UCB and contextualized PIV against other inflammation-based indices. Methods: We retrospectively analyzed 119 patients with histologically confirmed UCB who were treated between 2019 and 2024. PIV was calculated as (neutrophils × platelets × monocytes) ÷ lymphocytes. Additional indices included the NLR, SII, SIRI, and PLR. Progression-free survival (PFS) and overall survival (OS) were estimated using Kaplan–Meier analysis, and prognostic factors were assessed using Cox regression. Results: Among 119 patients (median age, 72 years; 88% male), 68 were diagnosed with NMIBC and 51 with MIBC. Elevated PIV levels were significantly associated with NMIBC progression to MIBC (*p* = 0.028) and strongly correlated with NLR, SII, SIRI, and PLR. Patients with high PIV exhibited shorter OS (24 vs. 45 months) and PFS (20 vs. 35 months) than those with low patients (*p* < 0.001). Although the prognostic value was evident in the univariate analyses, PIV did not retain significance in multivariate models. Conclusion: Elevated PIV levels predict adverse survival outcomes and progression in UCB, underscoring its potential as a cost-effective and accessible biomarker for risk stratification. Prospective validation in larger cohorts is warranted to confirm its role in personalized patient management.

## Simple Summary

Bladder cancer presents with substantial heterogeneity, ranging from less aggressive non–muscle-invasive forms to more advanced muscle-invasive disease. The identification of simple and reliable prognostic markers is critical for optimizing treatment strategies. In this study, we investigated the prognostic relevance of the pan-immune-inflammation value (PIV), a blood-derived index reflecting systemic inflammation and immune status. A cohort of 119 patients with bladder cancer was analyzed, revealing that elevated PIV levels were associated with accelerated disease progression and reduced survival. Although PIV did not retain independent prognostic significance after adjustment for other variables, it demonstrated potential as a cost-effective and readily available tool for risk stratification. Validation in larger, prospective cohorts is warranted to establish its clinical applicability.

## 1. Introduction

Urothelial carcinoma of the bladder (UCB) is the most prevalent malignancy of the urinary tract, and represents a substantial global health burden [1]. According to recent data from the GLOBOCAN database, bladder cancer ranks as the tenth most commonly diagnosed malignancy worldwide, with an estimated 570,000 new cases and approximately 200,000 cancer-related deaths annually [2]. The vast majority of bladder cancers originate in the urothelium, with the highest incidence rates observed in industrialized countries, particularly among elderly male populations [3,4]. Clinically, UCB exhibits considerable heterogeneity, encompassing a spectrum from non-muscle-invasive bladder cancer (NMIBC), which accounts for approximately 75% of initial diagnoses, to more advanced forms, including muscle-invasive bladder cancer (MIBC) and metastatic disease [3,5]. Despite significant advances in surgical techniques, intravesical therapies, systemic chemotherapy, immunotherapy, and targeted treatment modalities, disease recurrence and progression continue to pose major clinical challenges [6,7]. NMIBC is associated with a high recurrence rate, up to 70%, and carries a 10–20% risk of progression to MIBC [8]. In contrast, MIBC and metastatic UCB are linked to poor long-term prognosis, with five-year survival rates remaining below 50% and 10%, respectively [9].

Given this complex clinical trajectory, identification of robust prognostic biomarkers is essential for effective risk stratification, individualized treatment planning, and optimal surveillance strategies. In recent years, systemic inflammation- and nutrition-based indices derived from routine hematological parameters have garnered increasing interest for their potential to predict oncologic outcomes across a range of malignancies, including bladder cancer. Nevertheless, their prognostic relevance in UCB warrants further investigation using rigorously designed clinical studies.

The Pan-Immune-Inflammation Value (PIV) is an emerging composite biomarker derived from peripheral blood cell counts, specifically neutrophils, platelets, monocytes, and lymphocytes, and is designed to reflect the dynamic interplay between systemic inflammation and host immune competence [10]. Biologically, PIV encapsulates the contributions of key cellular elements that are actively involved in tumor-related inflammation, immunosuppression, and metastatic dissemination [11]. Increased neutrophil and monocyte levels have been associated with the facilitation of tumor proliferation and angiogenesis, predominantly mediated by the release of pro-inflammatory cytokines (e.g., interleukin-6 [IL-6], tumor necrosis factor-alpha [TNF-α]) and angiogenic growth factors, including vascular endothelial growth factor (VEGF) [12,13]. Concurrently, platelets play a pivotal role in facilitating immune evasion and enhancing the survival of circulating tumor cells by shielding them from natural killer (NK) cell-mediated cytotoxicity [14]. Conversely, lymphopenia is indicative of impaired anti-tumor immunity and reduced cytotoxic T-cell function [15].

By quantifying this immunological disequilibrium, PIV serves as a dynamic surrogate marker of tumor–host interactions and systemic inflammatory status. Its clinical appeal lies in its simplicity, cost-effectiveness, and accessibility given its derivation from routine complete blood counts. These attributes render PIV a practical tool for risk stratification and prognostic evaluation of a variety of malignancies. An expanding body of evidence has underscored the prognostic relevance of elevated PIV levels in several cancer types including non-small cell lung cancer [16], breast cancer [17], colorectal cancer [10], and pancreatic adenocarcinoma [18], wherein higher PIV scores have consistently been associated with unfavorable outcomes in terms of overall survival (OS), disease-free survival (DFS), and progression-free survival (PFS). These observations suggest that PIV may function not only as a prognostic indicator but also as a surrogate marker for tumor aggressiveness and systemic immune dysregulation, thereby warranting further validation in large-scale, tumor-specific cohorts.

This study aimed to comprehensively evaluate the association between PIV and clinical outcomes in patients with UCB. Additionally, we examined the relationship between PIV and other established prognostic indices, including the Neutrophil-to-Lymphocyte Ratio (NLR), Systemic Immune-Inflammation Index (SII), and Systemic Inflammation Response Index (SIRI). Elucidating the role of PIV and related inflammatory markers in UCB may facilitate risk stratification, particularly in early-stage disease, thereby supporting the optimization of treatment strategies and contributing to improved patient management and clinical outcomes.

## 2. Materials and Methods

### 2.1. Participant Selection & Collection of the Data & Follow-Up

This retrospective study included data from patients diagnosed with UCB who were collaboratively managed by the Department of Urology and Medical Oncology at the University of Health Sciences Antalya Education and Research Hospital (UHSAERH) between March 2019 and November 2024. All patients underwent radiological staging after histopathological confirmation of the diagnosis through maximal transurethral resection of the bladder tumor (TURBT). Patients diagnosed with NMIBC were managed with intravesical Bacillus Calmette-Guérin (BCG) therapy. In contrast, patients with MIBC received stage-appropriate standard treatments, including primary surgery followed by adjuvant therapy in operable cases, concurrent chemoradiotherapy for locally advanced disease, and a combination of immunotherapy and systemic chemotherapy for patients with advanced or metastatic disease. One week before the TURBT procedure, a complete blood count and comprehensive biochemical analyses were performed.

**Figure 1:**
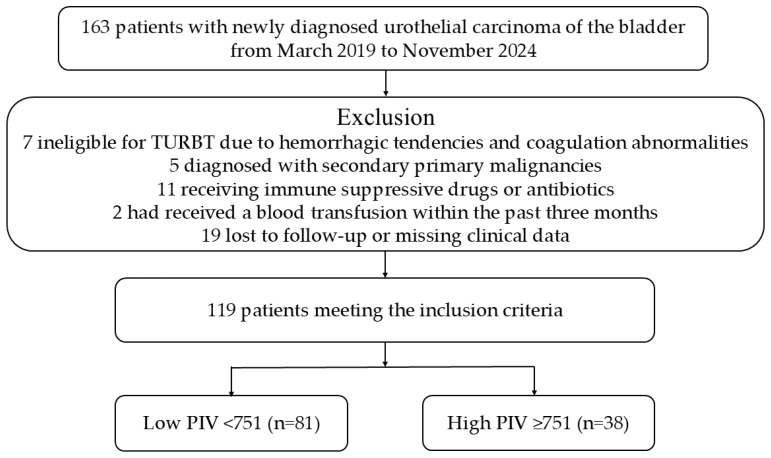
Flowchart of the study according to CONSORT diagram. Abbreviations: TURBT, transurethral resection of bladder tumor; PIV, pan-immune-inflammation value; CONSORT, consolidated standards of reporting trials.

Seven patients who were ineligible for TURBT due to bleeding or coagulation disorders, five patients diagnosed with a second primary malignancy, 11 patients receiving antibiotic therapy or immunosuppressive therapy for chronic comorbid conditions, two patients who had undergone blood transfusion within the past three months, and 19 patients who were lost to follow-up or had incomplete clinical data were excluded from the study. Ultimately, 119 patients with complete clinical, histopathological, and laboratory records were analyzed, as presented in the flowchart (Figure 1).

The dataset encompasses an extensive range of demographic, clinical, and treatment-related variables, including age, sex, Eastern Cooperative Oncology Group performance status (ECOG PS), comorbid conditions, smoking and alcohol history, intravesical BCG therapy, tumor-node-metastasis (TNM) stage, lymph node involvement, disease burden, adjuvant treatments, therapeutic response, disease progression, subsequent management strategies, and OS.

PIV was calculated using the following formula proposed by Fuca et al.: PIV = (neutrophils × platelets × monocytes)/lymphocytes [10]. For comparison, the following additional indices were computed: SII = NLR × platelets [19], SIRI = NLR × monocytes [20], platelet-to-lymphocyte ratio (PLR) = platelets/lymphocytes [21], and NLR = neutrophils/lymphocytes [22].

All patients were monitored according to internationally accepted guidelines. Follow-up consisted of physical examinations, laboratory analyses, and cystoscopic evaluations at 3–6 months intervals during the first two years, at 6–12 months intervals up to five years, and annually thereafter. Radiological imaging was conducted when clinically indicated, and the recurrence, progression, and survival outcomes were systematically documented. The primary endpoint of this study was OS, defined as the period between the date of pathological diagnosis and either death from any cause or the last follow-up assessment. The secondary endpoint was PFS, calculated from the date of histopathological diagnosis until the first documentation of disease progression, death, or the most recent follow-up.

### 2.2. Ethical Statement

The study was conducted in line with the ethical standards outlined in the Declaration of Helsinki (initially adopted in 1964 and most recently updated in 2024). The research protocol received approval from the Institutional Review Board of the University of Health Sciences Antalya Education and Research Hospital (Approval No: 2025-57). Because of its retrospective design, informed consent from patients was waived. To protect confidentiality, all patient data were anonymized prior to analysis.

### 2.3. Statistical Analysis

All statistical analyses were conducted using the Statistical Package for the Social Sciences (SPSS) software, version 27.0 for Windows (IBM Corp., Chicago, IL, USA). The distribution of continuous variables was examined with the Kolmogorov–Smirnov test. Variables with a normal distribution were reported as mean ± standard deviation, whereas non-normally distributed variables were expressed as median values with minimum and maximum ranges. The prognostic accuracy of PIV, NLR, SII, SIRI, and PLR in predicting mortality was analyzed through Receiver Operating Characteristic (ROC) curve analysis, and the most appropriate cut-off points were identified using the Youden index. Associations between PIV and categorical clinical characteristics were evaluated using the chi-square test or Fisher’s exact test, where appropriate. Correlations between group distributions were examined using Spearman’s rank correlation. Survival outcomes, including OS and PFS, were estimated using the Kaplan–Meier method, and differences between groups were assessed with the log-rank test. Variables associated with survival in univariate Cox regression analyses were subsequently entered into a multivariate Cox regression model. The potential for multicollinearity was assessed by calculating the Variance Inflation Factor (VIF) values for the inflammatory indices included in the regression model. The VIF values ranged from 1.8 to 4.5, and since none exceeded the threshold of 10, no significant multicollinearity was detected. Therefore, all variables were retained in the multivariate regression analysis. A *p*-value < 0.05 was considered statistically significant.

## 3. Results

The study cohort consisted of 119 patients diagnosed with UCB with a median age of 72 years (interquartile range [IQR]: 46–96). A total of 105 patients (88.2%) were male. Comorbidities were present in 89.0% of patients, a history of smoking was documented in 82.4% of patients, and alcohol consumption was reported in 10.9%. NMIBC was identified in 68 patients (57.1%), of whom 45 (66.2%) underwent intravesical BCG therapy. MIBC was diagnosed in 51 patients (42.9%), with lymph node involvement observed in 21 cases (41.2%) and distant metastases in 8 cases (15.7%). The sociodemographic and clinical characteristics stratified by PIV cut-off are presented in Table 1.

Among the 68 patients initially diagnosed with NMIBC, 19 progressed to MIBC during the serial clinical surveillance. Progression from NMIBC to MIBC was significantly more frequent in patients with elevated PIV levels than in those with lower PIVs (*p* = 0.028) (Table 1).

Smoking history, advanced disease stage, and elevated NLR, SII, SIRI, and PLR were significantly more common among patients with high PIV levels (*p* < 0.05) (Table 1). A strong positive correlation was observed between PIV and NLR (r = 0.688, *p* < 0.001), as well as with SII (r = 0.703, *p* = 0.001), SIRI (r = 0.497, *p* = 0.001), and PLR (r = 0.498, *p* < 0.001) (Table 2).

### 3.1. Cutoff Values of the Laboratory Parameters

**Figure 2:**
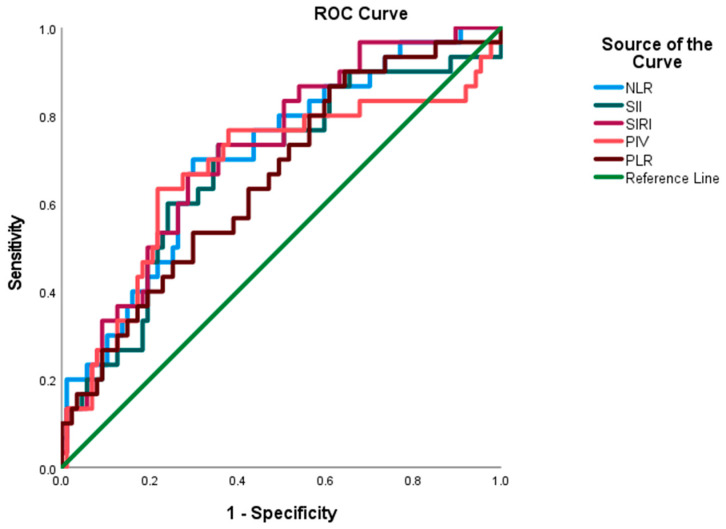
Comparison of the capability of the PIV, NLR, SII, SIRI, and PLR to predict mortality in patients with newly diagnosed urothelial carcinoma of the bladder with ROC curve analysis.

The prognostic utility of various immune-inflammatory indices in predicting mortality among patients with UCB was assessed using the ROC curve analysis (Table 3). The indices demonstrating the highest area under the curve (AUC) values were as follows: NLR, 0.71 (95% CI: 0.60–0.82); SII, 0.68 (95% CI: 0.56–0.79); SIRI, 0.72 (95% CI: 0.62–0.82); PLR, 0.66 (95% CI: 0.54–0.77); and PIV, 0.68 (95% CI: 0.55–0.80) (Figure 2). The optimal cut-off values, determined using the maximum Youden index, were 3.16 for NLR, 778 for SII, 1.26 for SIRI, 165 for PLR, and 751 for PIV (Table 3, Figure 2).

### 3.2. Survival Analysis

**Figure 3:**
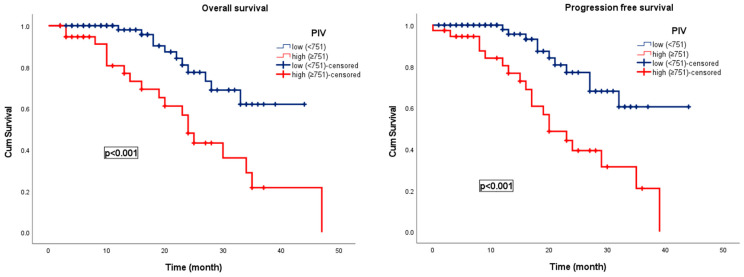
Kaplan–Meier survival curves stratified by the PIV in patients with newly diagnosed urothelial carcinoma of the bladder.

During a median follow-up period of 17.5 months, disease progression was observed in 33 patients (27.7%) and 21 patients (17.6%) died. In patients with UCB, the median OS was 39 months (95% CI: 28.1–49.9), and the median PFS was 28 months (95% CI: 24.3–32.7). In the low PIV group, the median OS and PFS were 45 months (95% CI: 32.6–58.1) and 35 months (95% CI: 31.5–39.6), respectively, while in the high PIV group, the median OS and PFS were 24 months (95% CI: 21.1–26.9) and 20 months (95% CI: 13.9–26.1), respectively. Patients with low PIV levels had significantly longer OS and PFS than those with elevated PIV levels (*p* < 0.001). Kaplan–Meier survival curves for OS and PFS stratified by PIV levels are presented in Figure 3.

In the univariate Cox proportional hazards analysis, smoking history, NLR, SII, SIRI, PLR, and PIV were significantly associated with OS (*p* < 0.05) (Table 4). Similarly, in the univariate analysis of factors potentially influencing PFS, NLR, SII, SIRI, PLR, and PIV were significantly associated with PFS (*p* < 0.05) (Table 4).

Collinearity diagnostics showed acceptable tolerance (0.21–0.42) and VIF values (2.38–4.72) for all inflammatory indices (PIV, NLR, SII, SIRI, PLR). Since none exceeded the critical VIF threshold of 10, no significant multicollinearity was detected, and all variables were retained in the multivariate regression analysis (Table 5).

However, in the multivariate Cox regression analysis, none of the evaluated prognostic indices showed a statistically significant association with clinical outcomes.

## 4. Discussion

The clinical heterogeneity characteristic of UCB is increasingly being recognized as a consequence of the intricate interplay between tumor-intrinsic factors and host immunological and inflammatory mechanisms [1]. Accumulating evidence suggests that systemic inflammation critically influences cancer initiation, progression, and immune escape by modulating both the local tumor microenvironment and the systemic host response [23]. Key cellular constituents of the systemic inflammatory response, including neutrophils, lymphocytes, monocytes, and platelets, exert their effects on tumor biology via the secretion of cytokines, growth factors, and pro-angiogenic mediators [24]. These factors collectively facilitate tumor proliferation, angiogenesis, and attenuation of anti-tumor immunity. Within this framework, composite indices derived from routinely available peripheral blood hematological parameters such as PIV may effectively capture the complex crosstalk between immune activation and systemic inflammation [10]. Accordingly, PIV represents a surrogate biomarker of tumor-promoting inflammation and immune dysregulation and has potential prognostic utility. Its application could inform therapeutic decision-making and enhance patient management through refined prognostic stratification.

The integrated assessment of neutrophils, monocytes, platelets, and lymphocytes, the cellular constituents of PIV, may offer a more comprehensive representation of the complex immune dysregulation associated with malignancy. Neutrophils and platelets are recognized for their protumorigenic functions [12,14], while lymphocytes are central to effective antitumor immune responses [15]; thus, the imbalance among these elements, as quantified by PIV, has significant prognostic implications. From a clinical perspective, PIV, derived from routinely available peripheral blood parameters, constitutes a practical, reproducible, and cost-effective biomarker with potential utility in the early risk stratification of patients with UCB and in informing personalized therapeutic approaches. Given the high rates of recurrence and progression in NMIBC, as well as the poor prognosis associated with advanced-stage disease, the incorporation of systemic inflammation-based markers such as PIV in clinical decision-making may improve prognostic precision beyond that afforded by conventional staging systems.

Recent studies have highlighted a strong association between PIV and cancer prognosis [10,16,17,18]. Elevated PIV consistently serves as a prognostic indicator of unfavorable outcomes across multiple clinical endpoints including treatment response, disease recurrence, and OS. Its prognostic significance extends beyond common cancers, such as lung, breast, and colorectal cancers [10,16,17], to rare malignancies, including pancreatic cancer, glioma, and hepatocellular carcinoma [18,25,26]. Notably, in non-metastatic MIBC, elevated PIV has been linked to poorer disease-free and overall survival, and has been identified as an independent risk factor for OS [27]. Beyond PIV, prior studies in bladder cancer focusing on other inflammation-based indices, particularly the SII and the SIRI, have similarly linked higher scores with adverse outcomes [28,29]. Although these reports evaluate different composite metrics than PIV, they converge on the same biological theme: systemic immune-inflammatory activation bears prognostic relevance in UCB.

The findings of this study revealed a significant association between elevated PIV levels and unfavorable clinical outcomes in patients with UCB. Patients in the high PIV group exhibited substantially shorter OS and PFS than those in the low PIV group. These results align with existing evidence from other malignancies, indicating that elevated PIV serves as a surrogate marker of heightened systemic inflammation and compromised host immune function, both of which are implicated in tumor progression and poor prognosis. Furthermore, the strong correlations observed between PIV and established inflammatory indices, including NLR, SII, SIRI, and PLR, further underscore the role of systemic inflammation in shaping the disease trajectory. While the prognostic significance of PIV was evident in univariate analyses, its loss of statistical significance in multivariate Cox regression may reflect the influence of confounding variables contributing to UCB progression as well as limitations related to sample size. Moreover, elevated PIVs were linked to an almost threefold increased risk of progression from NMIBC to MIBC, underscoring the potential utility of PIV as a clinically relevant indicator of tumor aggressiveness. Nevertheless, the inclusion of both non-muscle-invasive and muscle-invasive stages within a well-characterized cohort evaluated using comprehensive clinical, pathological, and laboratory parameters enhances the interpretability and clinical relevance of these findings.

In our analysis, PIV did not outperform other inflammation-based indices such as NLR, SII, SIRI, and PLR in terms of ROC and univariate Cox regression performance. This finding likely reflects the strong intercorrelation among these indices, as they are all derived from overlapping hematological parameters. To ensure the validity of our multivariate models, we performed additional collinearity diagnostics. VIF values for all indices ranged from 1.8 to 4.7, and tolerance values were between 0.21 and 0.42, all within accepted thresholds. These results indicated no evidence of significant multicollinearity, supporting the inclusion of all indices in the multivariate regression analysis. Nevertheless, the loss of independent significance for PIV in the multivariate model may reflect both the modest cohort size and the overlapping biological information shared among the indices. Importantly, PIV remains biologically compelling and clinically practical, as it integrates multiple cellular components into a single, readily available metric. Validation in larger, prospective cohorts is required to determine whether PIV can offer incremental prognostic value beyond existing indices.

This study has several important limitations. First, its retrospective and single-center design inherently introduces selection bias and restricts generalizability. Second, the relatively small cohort size limited the statistical power, particularly for multivariate analyses. Moreover, because enrollment occurred over several years, follow-up durations were not uniform; patients enrolled later inevitably had shorter follow-up times, which may have affected the accuracy of progression and survival estimates. Finally, heterogeneity in treatment approaches and the inclusion of both NMIBC and MIBC stages may have further confounded outcome interpretation. Despite these constraints, our study contributes valuable preliminary insights into the potential role of PIV in bladder cancer and underscores the need for larger, prospective, and multicenter studies with stage-specific stratification and standardized follow-up protocols.

## 5. Conclusions

In this exploratory pilot analysis, higher PIV was associated with shorter OS and PFS and with progression from NMIBC to MIBC; however, PIV did not demonstrate independent prognostic significance after adjustment. To establish the independent prognostic value of PIV, prospective, multicenter studies with stage-stratified and larger cohorts are required. Such studies would ensure an adequate number of events for robust and interpretable multivariable modeling, allow evaluation of the incremental value beyond existing indices (e.g., SII/SIRI), and enable the exploration of composite or integrative inflammation signatures.

## Figures and Tables

**Table 1:** Clinicopathological, sociodemographic, and laboratory data of patients with newly diagnosed urothelial carcinoma of the bladder classified according to the PIV.

Variables		PIV		*p*
		Low (<751) *n*, %	High (≥751) *n*, %	
Age	<65	18 (22.2)	4 (10.5)	0.098
	≥65	63 (77.8)	34 (89.5)	
Sex	Male	72 (88.9)	33 (86.8)	0.481
	Female	9 (11.1)	5 (13.2)	
ECOG PS	0–1	59 (89.4)	41 (77.4)	0.059
	≥2	7 (10.6)	12 (22.6)	
Comorbidity	No	7 (9.9)	6 (12.5)	0.688
	Yes	64 (90.1)	42 (87.5)	
Smoking status	No	9 (11.1)	12 (31.6)	0.008
	Yes	72 (88.9)	26 (68.4)	
Alcohol consumption	No	69 (85.2)	37 (97.4)	0.039
	Yes	12 (14.8)	1 (2.6)	
Intravesical BCG treatment	No	50 (61.8)	24 (63.2)	0.158
	Yes	31 (38.2)	14 (36.8)	
Transition from NMIBC to MIBC in clinical surveillance	No	31 (86.1)	18 (56.3)	0.028
	Yes	5 (13.9)	14 (43.7)	
Lymph node involvement	No	63 (82.9)	27 (77.1)	0.282
	Yes	13 (17.1)	8 (22.9)	
Distant metastasis	No	70 (92.1)	36 (94.7)	0.148
	Yes	6 (7.9)	2 (5.3)	
Tumor stage	pTa-Tis	13 (19.1)	6 (11.8)	0.005
	pT1	35 (51.5)	14 (27.5)	
	pT2	13 (19.1)	19 (37.3)	
	pT3–4	7 (10.3)	12 (23.5)	
NLR	<3.16	70 (86.4)	7 (18.4)	<0.001
	≥3.16	11 (13.6)	31 (81.6)	
SII	<778	63 (77.8)	1 (2.6)	<0.001
	≥778	18 (22.2)	37 (97.4)	
SIRI	<1.26	44 (54.3)	1 (2.6)	<0.001
	≥1.26	37 (45.7)	37 (97.4)	
PLR	<165	65 (80.2)	11 (28.9)	<0.001
	≥165	16 (19.8)	27 (71.1)	

Abbreviations: PIV, Pan-Immune-Inflammation Value; ECOG PS, Eastern Cooperative Oncology Group Performance Status; BCG, Bacillus Calmette-Guérin; NMIBC, Non-muscle-invasive Bladder Cancer; MIBC, Muscle-invasive Bladder Cancer; NLR, Neutrophil-to-Lymphocyte Ratio; SII, Systemic Immune-Inflammation Index; SIRI, Systemic Inflammation Response Index.

**Table 2:** The relationship between the prognostic immune inflammation markers in UCB.

		NLR	SII	SIRI	PLR	PIV
NLR	r	1.000	0.656	0.467	0.469	0.663
	*p*	-	<0.001	<0.001	<0.001	<0.001
SII	r	0.656	1.000	0.445	0.636	0.703
	*p*	<0.001	-	<0.001	<0.001	<0.001
SIRI	r	0.467	0.445	1.000	0.298	0.497
	*p*	<0.001	<0.001	-	0.001	<0.001
PLR	r	0.469	0.636	0.298	1.000	0.498
	*p*	<0.001	<0.001	0.001	-	<0.001

Abbreviations: PIV, Pan-Immune-Inflammation Value; NLR, Neutrophil-to-Lymphocyte Ratio; SII, Systemic Immune-Inflammation Index; SIRI, Systemic Inflammation Response Index; PLR, Platelet-to-Lymphocyte Ratio.

**Table 3:** Comparison of the AUC values for the PIV NLR, SII, SIRI, and PLR using ROC curve analysis.

	AUC	Std. Error	95% CI		*p*	Cut Off Value
			Lower Bound	Upper Bound		
NLR	0.709	0.054	0.603	0.815	0.001	3.16
SII	0.678	0.059	0.563	0.793	0.004	778
SIRI	0.721	0.052	0.619	0.823	0.001	1.26
PIV	0.675	0.064	0.550	0.800	0.004	751
PLR	0.656	0.057	0.543	0.768	0.011	165

Abbreviations: AUC, Area under the curve; Std, Standard deviation; CI, Confidence interval; NLR, Neutrophil-to-Lymphocyte Ratio; SII, Systemic Immune-Inflammation Index; SIRI, Systemic Inflammation Response Index; PIV, Pan-Immune-Inflammation Value; PLR, Platelet-to-Lymphocyte Ratio.

**Table 4:** Univariate Cox regression model of OS and PFS in patients with newly diagnosed urothelial carcinoma of the bladder.

(Univariate) Overall Survival					(Univariate) Progression Free Survival				
	HR	95.0% CI for HR		*p*			95.0% CI for HR		*p*
		Lower	Upper			HR	Lower	Upper	
Age	6.67	0.907	49.067	0.062	Age	6.063	0.825	44.579	0.077
Sex	0.697	0.165	2.935	0.623	Sex	0.719	0.171	3.031	0.653
ECOG PS	1.944	0.533	9.311	0.266	ECOG PS	2.251	0.689	8.112	0.314
Comorbidity	11.62	0.004	11.3	0.482	Comorbidity	22.058	0.007	733.483	0.455
Smoking	0.42	0.178	0.99	0.047	Smoking	0.5	0.213	1.176	0.112
Alcoholism	0.457	0.108	1.932	0.287	Alcoholism	0.358	0.083	1.539	0.167
Intravesical BCG treatment	1.911	0.863	4.232	0.11	Intravesical BCG treatment	1.777	0.805	3.923	0.155
Lymph node involvement	1.342	0.629	2.865	0.447	Lymph node involvement	1.542	0.726	3.274	0.26
Distant metastasis	0.61	0.183	2.036	0.421	Distant metastasis	0.644	0.192	2.155	0.475
Tumor stage	0.856	0.413	1.775	0.676	Tumor stage	0.863	0.405	1.838	0.702
NLR	2.263	1.079	4.747	0.031	NLR	2.114	1.015	4.401	0.045
SII	3.205	1.418	7.242	0.005	SII	3.238	1.437	7.295	0.005
SIRI	3.419	1.302	8.978	0.013	SIRI	3.238	1.236	8.482	0.017
PIV	3.3	1.556	6.998	0.002	PIV	3.533	1.676	7.448	0.001
PLR	2.505	1.196	5.245	0.015	PLR	2.374	1.135	4.967	0.022

Abbreviations: ECOG PS, Eastern Cooperative Oncology Group Performance Status; BCG, Bacillus Calmette-Guérin; NLR, Neutrophil-to-Lymphocyte Ratio; SII, Systemic Immune-Inflammation Index; SIRI, Systemic Inflammation Response Index; PIV, Pan-Immune-Inflammation Value; PLR, Platelet-to-Lymphocyte Ratio.

**Table 5:** Variance Inflation Factor (VIF) and Tolerance values of inflammatory indices.

	Tolerance	Variance Inflation Factor
PIV	0.42	2.38
NLR	0.36	2.77
SII	0.28	3.55
SIRI	0.21	4.72
PLR	0.25	3.98

Abbreviations: NLR, Neutrophil-to-Lymphocyte Ratio; SII, Systemic Immune-Inflammation Index; SIRI, Systemic Inflammation Response Index; PIV, Pan-Immune-Inflammation Value; PLR, Platelet-to-Lymphocyte Ratio.

## Data Availability

The datasets used in this study can be made available by the corresponding author upon reasonable request, with permission from the Urology Department of UHSAERH.

## Abbreviations

The following abbreviations are used in this manuscript:

UCB	Urothelial Carcinoma of the Bladder
NMIBC	Non-muscle-invasive Bladder Cancer
MIBC	Muscle-invasive Bladder Cancer
PIV	Pan-immune-inflammation Value
TNF	Tumor Necrosis Factor-Alpha
VEGF	Vascular Endothelial Growth Factor
PFS	Progression-free Survival
OS	Overall Survival
NLR	Neutrophil-to-lymphocyte Ratio
SII	Systemic Immune-inflammation Index
SIRI	Systemic Inflammation Response Index
PLR	Platelet-to-lymphocyte Ratio
TURBT	Transurethral Resection of the Bladder Tumor
ECOG PS	Eastern Cooperative Oncology Group Performance Status
BCG	Bacillus Calmette-Guérin
TNM	Tumor-Node-Metastasis
NK	Natural Killer
